# 

**Published:** 1894-01

**Authors:** 


ESTABLISHED 1855. SUBSCRIPTION, $2.00.

LIBRARY Index S. G. O.

### ATLANTA MEDICAL AND SURGICAL JOURNAL.

OLD SERIES, VOL. XXVI. NEW SERIES, VOL. X. } ATLANTA, GA., JANUARY, 1894. NO. 11.

LUTHER B. GRANDY, M. D., MANAGING EDITOR.

M. B. HUTCHINS, M. D., BUSINESS MANAGER.

